# Ultrafast, One-Step, Salt-Solution-Based Acoustic Synthesis of Ti_3_C_2_ MXene

**DOI:** 10.1021/acsnano.0c07242

**Published:** 2021-02-26

**Authors:** Ahmed El Ghazaly, Heba Ahmed, Amgad R. Rezk, Joseph Halim, Per O. Å. Persson, Leslie Y. Yeo, Johanna Rosen

**Affiliations:** † Department of Physics, Chemistry, and Biology (IFM) Linköping University, SE-581 83 Linköping, Sweden; ‡ Micro/Nanophysics Research Laboratory, RMIT University, Melbourne, Victoria 3000, Australia

**Keywords:** MAX phase, MXene, surface acoustic waves, synthesis, electrochemistry

## Abstract

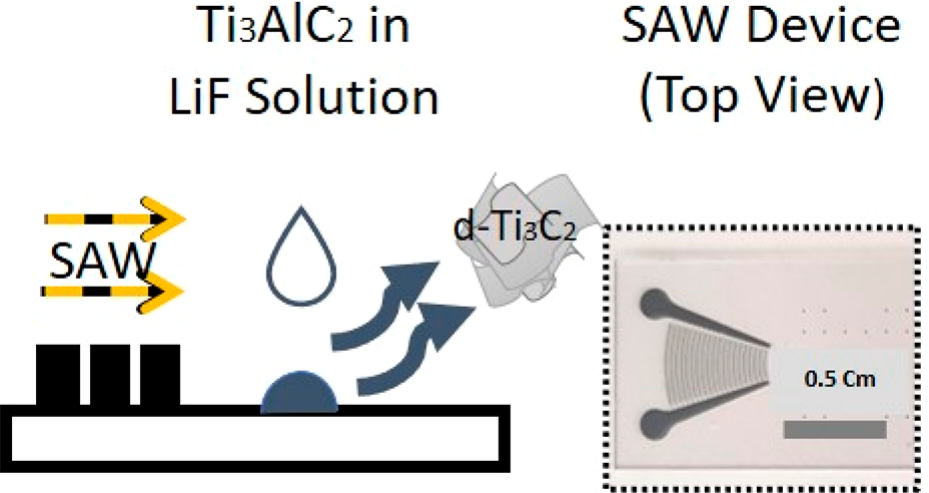

The current quest for two-dimensional transition metal carbides and nitrides (MXenes) has been to circumvent the slow, hazardous, and laborious multistep synthesis procedures associated with conventional chemical MAX phase exfoliation. Here, we demonstrate a one-step synthesis method with local Ti_3_AlC_2_ MAX to Ti_3_C_2_T_*z*_ MXene conversion on the order of milliseconds, facilitated by proton production through solution dissociation under megahertz frequency acoustic excitation. These protons combined with fluorine ions from LiF to selectively etch the MAX phase into MXene, whose delamination is aided by the acoustic forcing. These results have important implications for the future applicability of MXenes, which crucially depend on the development of more efficient synthesis procedures. For proof-of-concept, we show that flexible electrodes fabricated by this method exhibit comparable electrochemical performance to that previously reported.

Two-dimensional (2D) materials have gained significant attention in the past decade, as they display properties drastically different from their 3D precursors.^1−3^ Significant efforts have been devoted to control the procedures for synthesizing these materials and their resultant quality, so as to further enhance their properties for a variety of applications. MXenes are a more recent addition to the family of 2D materials,^4,5^ comprising 2D transition metal carbides and nitrides produced by selective etching of their parent 3D materials, the so-called M_*n*+1_AX_*n*,_ (MAX) phases, where M is a transition metal, A is an A-group element, and X is carbon and/or nitrogen (*n* = 1–3).^6^ The general MXene formula is consequently M_*n*+1_X_*n*_T_*z*_, where T represents surface termination groups (such as O, OH, F and/or Cl) and *z* is the number of groups per formula unit.^7,8^ Despite their young age, MXenes have already shown an outstanding potential for energy storage with the highest capacitance recorded for a 2D material^9−11^ and electromagnetic shielding with a shielding efficiency 30% higher than the previous record obtained for Al foil,^12^ among other applications. Additionally, MXenes are both conductive and hydrophilic, allowing for coassembly with polar species and enabling sustainable, green processability.^4,5^

MXenes are typically synthesized by time-consuming and laborious multistep procedures involving chemical etching and exfoliation of the atomically laminated parent materials. The conventional method for selective etching of the A-layer was developed by Nagiub et al., using hydrofluoric acid (HF) as the etchant for selective removal of Al, followed by intercalation with DMSO for delamination of the MXene multilayers.^4,5,13^ Two years later, Ghidiu et al. developed a method based on milder chemicals, LiF and HCl.^9^ Both of these methods are time-consuming, requiring at least 24 h, excluding the time needed for repeated washing processes. The latter is based on repeated centrifugation with distilled water to achieve a safe pH (∼5–6). Moreover, the processes require tedious and hazardous HF waste management procedures. More recently, modified MXene synthesis processes have been developed, which include chemical recipes combining HF with other acids, such as HNO_3_,^14^ or by etching with molten salt (ZnCl_2_).^15^ In the latter, a high-temperature and inert environment (such as Ar gas) is needed to prevent oxidation during the reaction, HCl is required to remove the Zn, and use of an intercalant is necessary to produce single flakes.^15^ In any case, all of the reported methods to date warrant the use of an acid at a certain stage in the MXene synthesis, and the fastest process takes about 5 h excluding washing and delamination.^16^

As such, there is a compelling need for acid-free and time efficient synthesis methods, which we address in the present work by exploiting the nonlinear electromechanical coupling afforded by megahertz (MHz) order surface-localized vibrations in the form of surface acoustic waves (SAWs). SAWs are 10 nm amplitude MHz order Rayleigh waves which are generated, confined to, and subsequently propagate along the surface of single crystal piezoelectric substrates. Because of their very high (∼10^8^ m s^–2^) surface acceleration, they have been shown to be a powerful yet efficient source for driving a myriad of microfluidic actuation and manipulation, while enabling the possibility for miniaturization given the chip scale operation.^17^ Beyond purely mechanical effects, it has recently been realized that the strong electromechanical coupling inherent in the piezoelectric substrate can drive dynamic polarization atomic- and molecular-scale phenomena that could, for example, result in ionization and exciton transport in 2D transition metal dichalcogenides to affect bandgap modulation^18^ or the exfoliation of their bulk crystals into single- and few-layer nanosheets.^19^ In addition, we have also found that the strong 10^8^ V m^–1^ evanescent electric field accompanying the SAW electromechanical coupling is able to drive molecular orientation in bulk crystals,^20,21^ as well as to induce dissociation of pure water to facilitate the production of free radicals in the absence of any catalysts.^22^ Here, we demonstrate MXene synthesis involving local ultrafast (millisecond) MAX to MXene conversion without any supply of acids by exploiting SAWs in the presence of a low concentration solution of LiF (∼0.05 M). More specifically, exposure of an aqueous mixture of the MAX phase with LiF to the SAW leads to proton production and increased diffusion of fluorine ions needed to etch Al from the MAX phase, in addition to accelerating the reaction kinetics. We prove the existence of individual MXene sheets dispersed in water with delamination facilitated by the SAW forcing, and filter MXene films for characterization. Altogether, these results articulate a strategy for MXene synthesis while retaining a comparable yield to conventional (mild) methods. Given that the low-cost chip scale SAW device is amenable to parallelization, the proof-of-concept demonstrated in this work could potentially facilitate future large-scale MXene synthesis.

## Results and Discussion

**Figure 1:**
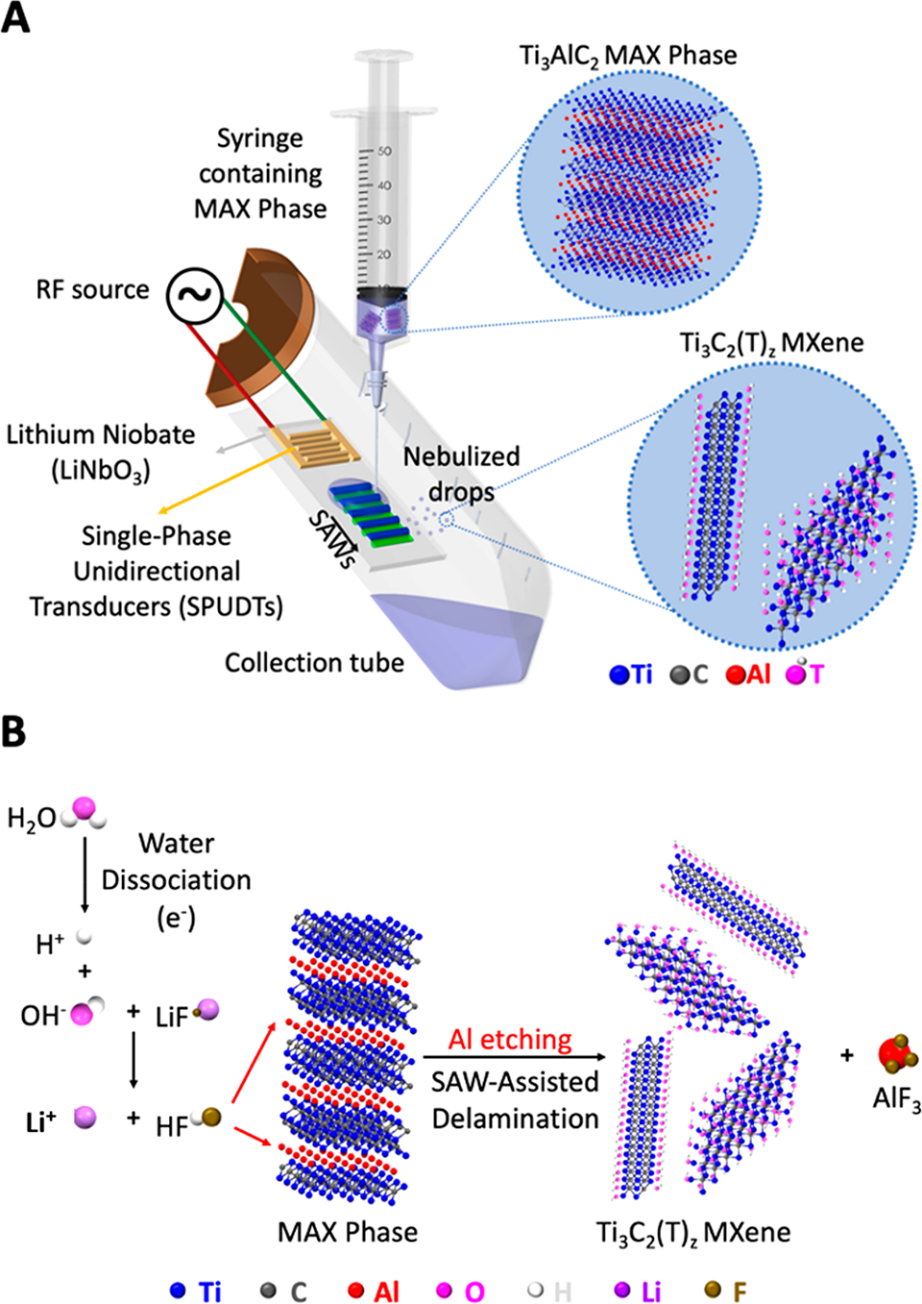
Schematic representation of the experimental setup and underlying physiochemical mechanism responsible for the SAW-facilitated derivation of Ti_3_C_2_T_*z*_ MXene. (A) SAWs are generated by applying an input sinusoidal electrical signal at the resonant frequency (30.5 MHz) to single phase unidirectional transducers (SPUDTs) patterned on the piezoelectric substrate (LiNbO_3_), that is, the SAW device. The SAWs are employed to drive the nebulization of liquid drops containing the MAX phase, dispensed manually *via* a syringe onto the substrate, to produce aerosol droplets containing the delaminated MXene that are subsequently collected within the collection tube enclosing the device. (B) The large evanescent electric field accompanying the SAW field drives dissociation of the water molecules within the drop containing the MAX phase to generate hydroxyl free radicals and protonated species, which in the presence of LiF combine to produce localized “*in situ* HF” that selectively etches away the Al in the Ti_3_AlC_2_ MAX phase. Subsequent delamination of the MXene sheets then occurs under the strong mechanical vibration associated with the SAW, whose acceleration on the substrate surface is on the order 10^8^ ms^–2^.

Synthesis of Ti_3_C_2_T_*z*_ MXene is facilitated by surface-localized vibrations in the form of SAWs in the presence of a fluoride-containing chemical. The schematic in Figure 1A shows the experimental setup employed in which the SAW energy is coupled into a sessile drop comprising 3D MAX phase particles dispersed in distilled water to which a small amount of LiF is added (0.26 g in 20 mL of deionized water). About 10% of the added LiF dissolves (the solubility of LiF in water at 25 °C is 0.026 g).^23^ The large (10^8^ V m^–1^) evanescent electric field of the SAW^22^ drives dissociation of the water in the sample to generate protons and hydroxyl radicals (eq 1)
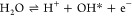
1The protons slightly decrease the pH of the solution momentarily from 6.8 to 5.5 which in turn increases the dissociation of the excess LiF, providing more F ions to react with the Al in the Ti_3_AlC_2_ MAX phase to produce Ti_3_C_2_T_*z*_ MXene and aluminum fluoride hydrate (AlF_3_·*x*H_2_O), which is soluble in water (eqs 2 and 3)
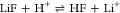
2
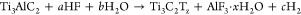
3Lithium ions are intercalated between the Ti_3_C_2_T_*z*_ layers in addition to water which causes an increase in the interlayer spacing, as shown with conventional etching procedures.^24^ Furthermore, the OH* free radicals may contribute to the formation of OH surface termination groups.

The high localized mechanical vibrations with acceleration of the order 10^8^ m s^–2^ on the substrate surface lead to an increase in the diffusion of ions in addition to an acceleration of the kinetics of the reaction causing localized etching of the MAX phase particles landing on the device. This consequently leads to spontaneous delamination of Ti_3_C_2_T_*z*_, resulting in delaminated Ti_3_C_2_ (*d*-Ti_3_C_2_T_*z*_) single sheets suspended in water. It should be noted that the input and output of the process are acid-free, since the input comprises salt (LiF), MAX phase, and water, whereas the output comprises MXene, the residual MAX phase, and LiF particles. The “*in situ* HF” that forms during the reaction is depleted and the pH of the solution returns to its original value after the process is terminated. This renders the technique to be the safest to date compared to the other reported techniques for producing MXene. More information on the safety of the present technique can be found in the SI.

**Figure 2:**
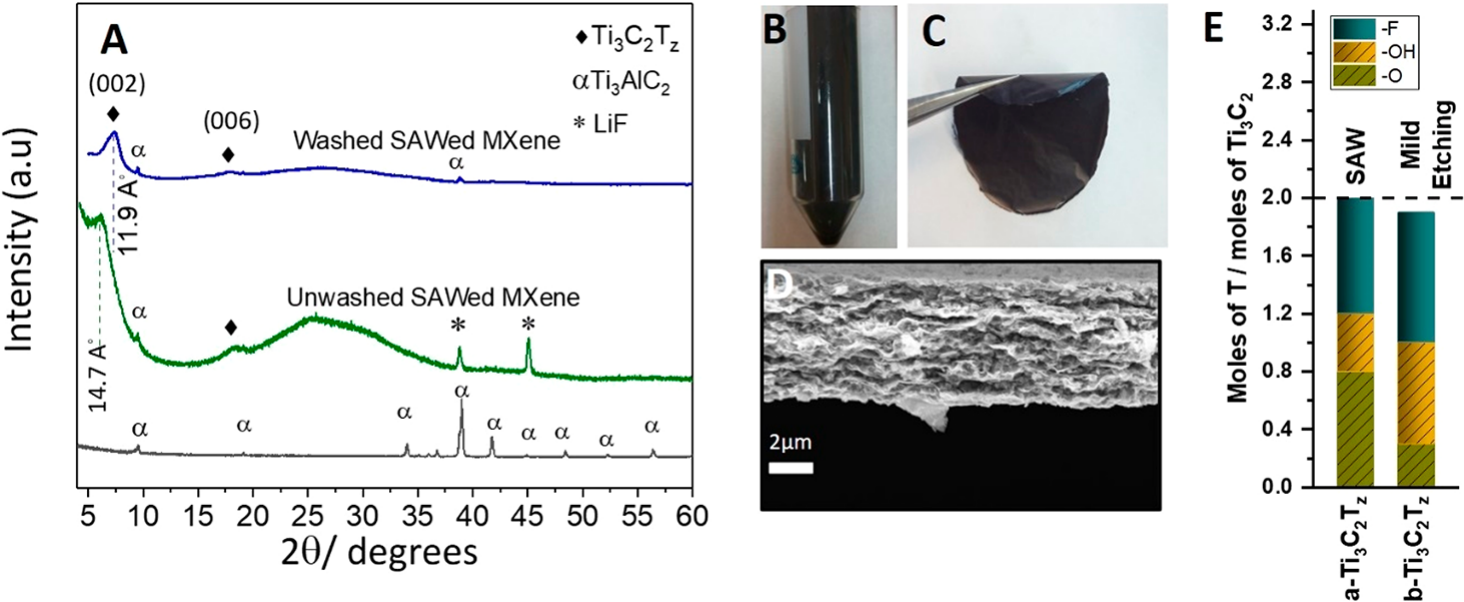
Analysis of Ti_3_C_2_T_*z*_ MXene obtained *via* SAW delamination. (A) XRD patterns for the Ti_3_AlC_2_ MAX phase (bottom, in black), MXene freestanding film (middle, in green), and MXene freestanding film after HCl and LiCl treatment to remove LiF residuals (top in blue). (B) MXene suspension produced by the SAW (20 V_rms_, 30.5 MHz) applied in the presence of LiF, which is filtered to produce (C) a flexible freestanding film comprising the delaminated MXene, here 34 mm in diameter. (D) Cross-section SEM image of the electrode of thickness 5 μm. (E) Results of the XPS analysis showing the number of moles of T (surface terminations) per Ti_3_C_2_T_*z*_ formula unit for the freestanding films: a-Ti_3_C_2_T_*z*_ (obtained *via* SAW delamination) and b-Ti_3_C_2_T_*z*_ (obtained *via* the conventional LiF + HCl method);^27^ note that if one termination is assumed per surface M atom, then the theoretical T_*z*_ number per formula is 2, as indicated by the horizontal dashed line. The average error for the surface termination values is ±0.1 mol.

X-ray diffraction (XRD) patterns are shown in Figure 2A for the Ti_3_AlC_2_ MAX phase (bottom, in black) and a Ti_3_C_2_T_*z*_ MXene freestanding film (middle, in green). Upon application of the SAW, the (002) peak of the Ti_3_AlC_2_ MAX phase is shifted from 2θ = 9.5° to 6°, indicating an expansion in the interlayer spacing, “*d”* = *c*/2, of about 4.5 Å. This expansion is due to the replacement of the Al layer with surface termination groups in addition to the intercalation of one layer of water and Li^+^ ions.^24,25^ The diffraction peaks observed at 38.7° and 44.8°, on the other hand, can be attributed to LiF. The residual amount of LiF can be removed by washing with 1 M HCl followed by 1 M LiCl.^26^ For comparison, the top in blue XRD pattern in Figure 2A is for a freestanding film filtered after HCl and LiCl washing, which shows the removal of LiF, and a decrease in the interlayer spacing from 14.7 to 11.9 Å, which in turn indicates the removal of the intercalated water layer. However, this latter procedure increases the synthesis time by approximately one more hour. Since one objective of the present project is to reduce the synthesis time, we chose here to report the characterization and electrochemical properties for the sample with the least synthesis time, that is, without HCl and LiCl washing. Figure 2B therefore shows the MXene suspension obtained after the one-step MXene synthesis facilitated by the SAW, and the resulting electrode obtained after filtering is shown in Figure 2C. The high flexibility of the pristine MXene electrode, whose cross-section is shown in Figure 2D, is evident.

To evaluate and quantify the MXene surface terminations, we performed X-ray photoelectron spectroscopy (XPS) measurements on the freestanding film (further details of the analysis can be found in Figure S2 and Tables S1, S2, and S3) from which we obtained the overall chemical composition (Table S1). The analysis shows removal of Al along with the resulting chemical formula for the freestanding MXene film: Ti_3_C_2_O_0.8_(OH)_0.4_F_0.8_.0.2H_2_O_ads_. Figure 2E plots the molarity of the surface termination groups for the SAW delaminated MXene and a conventional *in situ* LiF + HCl etched MXene,^27^ showing that the (O + OH)/F ratios are similar (60% [O + OH] vs 52%).

**Figure 3:**
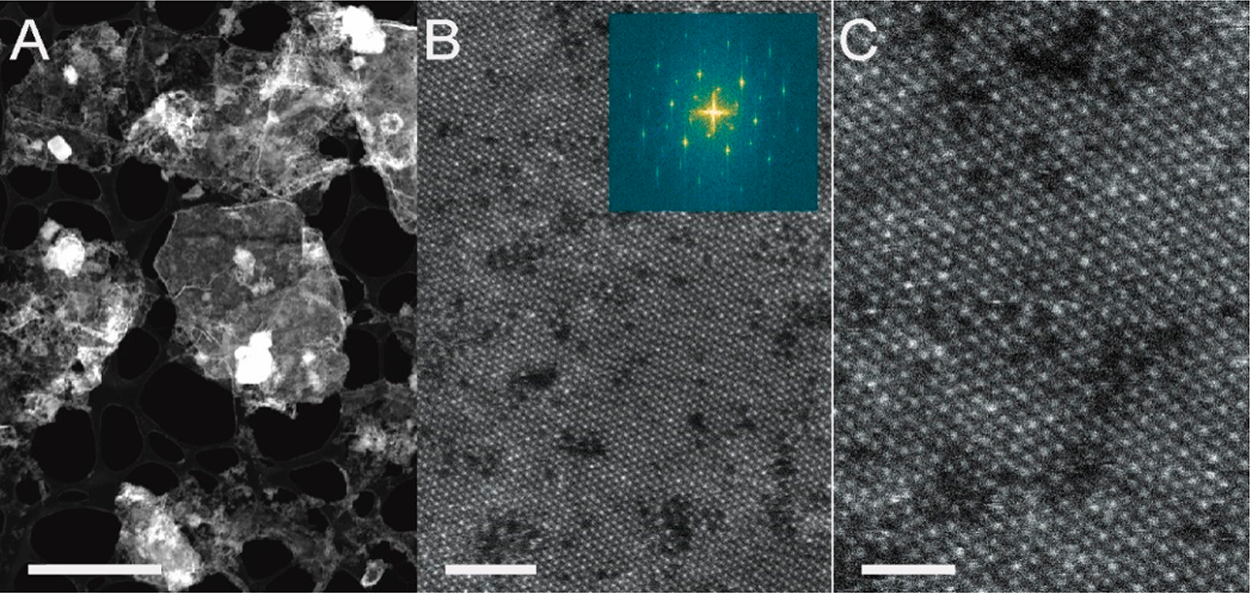
HAADF-STEM imaging of the SAW delaminated MXene showing (A) the layered structure of the uniformly thick MXene sheets, which under high-magnification reveals (B) local vacancies and (C) individually resolved atomic columns. Scale bars are 2 μm, 2 nm, and 1 nm, respectively.

The material was further examined by scanning transmission electron microscopy (STEM) as shown in Figure 3. The low-magnification image shown in Figure 3A reveals that the material exhibits a two-dimensional nature, as seen by the extended areas of uniform contrast that represent single or multiple stacked layers, with sheet sizes of up to 1 μm. Additional TEM analysis showing sheets of a size of about 1 μm is shown in Figure S3. In addition, it is also possible to observe local agglomerations of nanoscale particles that appear as bright clouds in the image. The lattice resolved structure from the same area is shown in Figure 3B in which the hexagonally close packed structure of Ti_3_C_2_T_*z*_ is clearly visible, though the structure also contains vacancies (dark spots) and vacancy clusters (dark irregular patches). The fact that we do not see any structure under the observed vacancies, not even under the small clusters of vacancies, shows that we have single layer sheets. Figure 3C shows the same image at higher magnification, revealing the individual atomic columns. Additionally, the sheet is locally identified to exhibit surface terminations, as evident from the apparently sparsely close-packed bright atoms across the sheet given that the column intensity is highly dependent on the local sheet alignment in relation to the electron beam.^28^

To demonstrate that the present technique for MXene synthesis provides a material with the same characteristics as those of conventionally synthesized MXene, we produced an electrode for initial analysis with respect to electrochemical energy storage (see Supporting Figure S4, S5, and Table S4). In particular, the cyclic voltammogram profiles of the Ti_3_C_2_T_*z*_ electrode show a capacitive charging mechanism with reversible redox peaks, which is consistent with that previously reported.^29^ Furthermore, the measured conductivity and volumetric capacitance, ∼1100 Sm cm^–1^ and 470 F cm^–3^ at 2 mV s^–1^, respectively, fall within the range reported previously for different etching/delamination protocols for this material.^30,31^ Furthermore, Figure S6 shows a comparison between the electrochemical performance of films from unwashed colloidal solutions obtained from mild conventional etching and that obtained with the SAW technique, showing a maximum of 14% difference in capacitance.

The present SAW technique is expected to have similar applicability toward the production of MXene using other F-containing chemicals in the form of a dispersion solution for the MAX phase, such as salts (NaF, KF). In other words, the protonation and highly localized mechanical acceleration of the SAW device combined with F ions etches the A layer in the MAX phase, to form MXene. The concentration may vary based on the solubility limits of the F-containing structure and the MAX phase particle size and concentration, although the amount of chemicals required can likely be tuned such that a higher yield can be achieved while keeping any F-containing byproduct to a minimum. The localized HF formed *in situ* is completely consumed in the etching process. This can also in principle be achieved by reducing the amount of LiF concentration to 0.05 M (solubility limit of LiF in water at 25 °C) and adjusting the amount of MAX phase so that all Al from the MAX is selectively etched, forming AlF_3_ hydrates.

Importantly, the present process uses only salt (LiF) and water as input, which eliminates the use of harmful acids or bases, and is attractive in terms of the limited equipment required, its ease of operation, and minimized waste management, as compared to conventional MXene synthesis methods. In addition, the product is safe to handle as the solution only temporally attains a pH value down to 5.5 upon SAW exposure, and then recovers to a value of 6.8 within several minutes after relaxation of the SAW. The obtained MXene sheets are stable and negatively charged with a −32 mV Zeta potential (see Figure S7). Moreover, the entire procedure is fast in the present work reducing the synthesis time of the MXene required to make an electrode (Figure 2C) from a minimum of 24 h with conventional synthesis methods to just 40 min in total for a yield (approximately 12%) that is comparable to that obtained *via* conventional methods. It should be stressed that the results presented in this work were attained with just a single SAW device. Given that the devices are produced through conventional wafer-scale photolithography, the method therefore has potential for upscaling through massive parallelization by exploiting the economies of scale associated with mass nanofabrication where a single device typically costs around US $1 (see further details in the SI).

## Conclusion

In summary, we report an ultrafast 3D to 2D conversion with an environmentally friendly, salt-based method for the synthesis of Ti_3_C_2_T_*z*_MXene. Given our previous observations of SAW-driven water splitting, we postulate that the protonation and production of fluorine ions from LiF, combined with the large mechanical forces associated with the SAW substrate undulation, when subjected to the MAX phase particles facilitates selective etching of Al and subsequently results in MXene delamination. The produced Ti_3_C_2_T_*z*_ MXene was confirmed by XRD, SEM, TEM, and XPS. In particular, we proved the existence of individual MXene sheets dispersed in water and filtered flexible MXene electrodes for characterization with respect to structure, composition and electrochemical behavior. The obtained results are consistent with that reported in the literature, therefore validating the technique as a viable synthesis method. Altogether, these results articulate a strategy for reducing MXene synthesis from a minimum of 24 h typically required using conventional methods, to an ultrafast conversion (approximately milliseconds) that for the present electrode production culminates in a total synthesis time of around 40 min with a single device.

## Materials and Methods

### Ti_3_AlC_2_ MAX Phase Preparation

The Ti_3_AlC_2_ MAX phase was synthesized by mixing TiC (∼2 μm particle size, 99.5% purity), Ti (<44 μm particle size, 99.9% purity), and Al (200 mesh size, 99% purity) powders (Alfa Aesar GmbH & Co KG, Karlsruhe, Germany) for 5 min in an agate mortar and pestle. The TiC/Ti/Al molar ratio was 2:1:1. The mixed powder was loaded in an alumina crucible and inserted in a tube furnace (MTI1700, MTI Corp, Richmond, CA, U.S.A.). The furnace was first pumped down and purged with Ar twice and then filled with Ar gas. The powder was heated up with a rate of 5 °C min^–1^ to 1450 °C, at which it was held for 2 h, prior to being cooled to 50 °C at a rate of 5 °C min^–1^. The slightly sintered MAX phase that was obtained was crushed using an agate mortar and pestle until an average particle size of 35 μm was attained.

### Device Fabrication and Operation

The SAW device consisted of a 128°-rotated Y-cut X-propagating single-crystal lithium niobate piezoelectric substrate (Roditi Ltd., London, U.K.) on which a focusing-elliptical single-phase unidirectional transducer (SPUDT) comprising 30 electrode finger pairs with an eccentricity of 0.83 was patterned using standard photolithographic processes. A primary function generator (SML01; Rhode & Schwarz, North Ryde, NSW, Australia) was used to generate a sinusoidal electric signal at an applied voltage of 20 V_rms_, which was subsequently amplified using a high-frequency 5 W amplifier (LYZ-22+, Mini Circuits, Brooklyn, NY, U.S.A.) and applied to the SPUDT to generate a uniform SAW whose frequency *f* is related to its wavelength λ through *f* = *c*/λ, wherein *c* = 3995 m s^–1^ is the speed at which the SAW propagates in LiNbO_3_. The input sinusoidal signal to the amplifier was set at a frequency *f* = 30 MHz to match the resonant frequency of the SPUDT, as determined by the width and spacing of its fingers (equal to λ/4 wherein λ = 132 nm at 30 MHz).

### MXene Synthesis

First, 0.26 g of LiF (LiF; 98+%, Sigma-Aldrich) in 20 mL of deionized Milli-Q water (18.2 MΩ cm, Merck Millipore, Bayswater, VIC, Australia, pH 6.8) was transferred to a hot stirring plate, stirred for 15 min at 80 °C then left to cool down to room temperature. Afterward, 100 mg of the Ti_3_AlC_2_ MAX phase was added to the LiF + water mixture to create a 5 mg mL^–1^ MAX phase suspension. The reason for adding a LiF amount higher than the solubility of LiF in water (∼0.05 M at 25 °C) is to create a F ion reservoir, since the dissolved amount of LiF does not provide the sufficient amount of F ions theoretically needed to etch all of the Al in Ti_3_AlC_2_ forming Ti_3_C_2_T_*z*_ (which is 0.08 M of LiF). The suspension of Ti_3_AlC_2_ + LiF was first homogenized using a vortex homogenizer and then dispensed manually using a syringe onto the surface of the device at the focal point of the SAW as a sessile drop. Upon activation of the SAW, the drop is immediately nebulized into aerosol droplets that contained the delaminated Ti_3_C_2_T_*z*_ sheets. The entire setup is enclosed within a collection tube to prevent loss of the aerosols and to facilitate ease of collection, 5 mL of which was directly used for transmission electron microscopy (TEM) and electron energy loss spectroscopy (EELS). For electrode production, a 15 mL suspension was sonicated for 30 min under N_2_ flow to improve their dispersion, followed by centrifugation for 10 min at 3000 rpm. The supernatant was used to prepare a film of 34 mm diameter and 5 μm thickness for further analysis. The film was prepared by vacuum filtering the suspension of 15 mL through a nanopolypropylene membrane (3501 Coated PP, 0.064 μm pore size, Celgard, U.S.A.). The film was dried in air overnight with a resulting weight of 8.6 mg. For electrochemistry measurements, a 2 μm film was prepared *via* the same procedure. For the removal of the residual LiF, the following procedure was used: Before sonication, the suspension was washed through 3 cycles of 40 mL of 1 M HCl (Fisher, technical grade) followed by 3 cycles of 40 mL of 1 M LiCl (Alfa Aesar, 98+%). Initially the suspension was centrifuged at 5500 rpm for 5 min, the supernatant of clear water was decanted, and then the washing cycles started. After each washing cycle, the suspension is centrifuged at 5500 rpm for 1 min and the supernatant is decanted. Then, the sample was washed through 6 cycles of 40 mL of distilled water. After washing, 15 mL of distilled water was added and deaerated by bubbling N_2_ gas through it which then was sonicated using an ultrasonic bath for 30 min while bubbling N_2_ through the suspension. The resulting suspension was centrifuged 10 min at 3000 rpm.

### Materials Characterization

The Ti_3_AlC_2_ MAX phase was analyzed by X-ray diffraction (XRD), using a powder diffractometer (Rigaku SmartLab, Wilmington, DE, U.S.A.) with Cu Kα radiation (λ = 1.54 Å) and a step size of 2θ of 0.01° s^–1^. Scanning electron microscopy (SEM) was performed on a Zeiss Supra 50 VP (Carl Zeiss SMT AG, Oberkochen, Germany). The XPS measurements were performed on a freestanding Ti_3_C_2_T_*z*_ film (Kratos AXIS Ultra^DLD^, Manchester, U.K.) under monochromatic Al-*K*_*α*_ (1486.6 eV) radiation. The sample was mounted using carbon double tape and clamped on the sample holder and grounded with a copper strip. The X-ray beam with a spot size of 300 × 800 μm irradiated the surface of the sample at an angle of 45° with respect to the surface ray. The electron analyzer received photoelectrons perpendicular to the surface of the sample with an acceptance angle of ±15°. XPS spectra were recorded for the following regions: Ti 2p, C 1s, O 1s, F 1s, Cl 2p, Al 2p, and Li 1s (the Cl 2p and Al 2p regions are not shown here and the quantification of Al was below the instrument detection limit) using a pass energy of 20 eV with a step size of 0.1 eV. The binding energy (BE) scale of all XPS spectra was referenced to the Fermi-edge (*E*_F_), which was set to a BE of 0 eV. Peak fitting for the high-resolution spectra was performed using CasaXPS Version 2.3.16 RP 1.6. Prior to peak fitting, the background contributions were subtracted using a Shirley function. For the Ti 2p_3/2_ and 2p_1/2_ components, the area ratios of the peaks were constrained to be 2:1, respectively. The colloidal stability was assessed by measuring its zeta potential (Malvern Zetasizer Nano S, Malvern, U.K.).

High-angle annular dark-field scanning transmission electron microscopy (HAADF-STEM) was performed in the Linköping double-corrected, monochromated FEI Titan^3^ 60-300 (FEI, Hillsboro, OR, U.S.A.) operated at 300 kV. Imaging was performed with a beam convergence semiangle of 22 mrad and a camera length of 0.185 m. The beam current during imaging was ∼20 pA. Samples for STEM were prepared by dispersing the ready material on a lacey carbon TEM grid.

### Electrochemical Characterization

All electrochemical measurements were performed in a three-electrode plastic Swagelok cell, with the prepared 2 μm (±0.15 μm), 25 μg, Ti_3_C_2_T_*z*_ film serving as the working electrode. The average thickness of the Ti_3_C_2_T_*z*_ film was calculated from seven different measurements from the cross-sectional area. The activated carbon (AC) serving as the counter electrode was prepared by mixing 95 wt % of YP-50 AC, supplied by Kuraray (Tokyo, Japan), and 5 wt % of polytetrafluoroethylene (PTFE) binder. The AC electrode was rolled up to form a film of ∼150 μm thickness and the weight was ∼3 mg. An Ag/AgCl electrode immersed in 1 M KCl was utilized as a reference electrode, and glassy carbon electrodes were used as current collectors. Electrochemical measurements were performed using a VSP potentiostat (BioLogic, Seyssinet-Pariset, France). Cyclic voltammetry (CV) was performed at scan rates from 2 to 1000 mV s^–1^. The voltage window was from −0.6 to +0.3 V and chosen based on the following steps: (a) The open circuit potential (OCP) was assigned as the upper positive potential to prevent the MXene from being oxidized. (b) Ten successive CV cycles at 20 mV s^–1^ were performed to assign the minimum voltage potential. The gravimetric capacitance *C*_g_ was calculated by integrating the discharging current according to
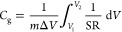
where *m* is the working electrode mass, Δ*V* is the voltage window, and SR is the scan rate. The electrode density ρ was 2.7 g cm^–3^ and the volumetric capacitance is obtained from *C*_v_ = ρ*C*_g_. Electrochemical impedance spectroscopy (EIS) with 10 mV amplitude was performed at the open circuit potential (OCP) between 10 mHz and 200 kHz. A set of charge/discharge (GCPL) tests were performed at 1, 2, 3, 5, and 10 A g^–1^. Stability tests were performed by galvanostatic charge/discharge (GCPL) at 10 A g^–1^ for 10 000 cycles.

## Supporting Information Available

Additional experimental results, including MXene characterization; XPS spectra with fitting curves for the freestanding film, table of the atomic percentage from the XPS; electrochemical characterization results in figures and supplementary text, table of specific capacitance for the freestanding films, SEM image for the film morphology, TEM images for Ti_3_C_2_ single flakes, zeta potential and Tyndall effect measurements for the Ti_3_C_2_ colloidal sheets (PDF)
