# Supplementary material for: Ultrafast, One-Step, Salt-Solution-Based Acoustic Synthesis of Ti3C2 MXene
Source: ACS Nano. 2021 Feb 26;15(3):4287–93. doi: 10.1021/acsnano.0c07242 (PMC8034768; doi:10.1021/acsnano.0c07242)
Supplement: Supplementary file 1 — nn0c07242_si_001.pdf [file nn0c07242_si_001.pdf]

Supporting Information

Title: Ultrafast, One-Step, Salt-Solution-Based Acoustic Synthesis of Ti_3C_2 MXene

Ahmed El Ghazaly [§‡], *Heba Ahmed* [£‡], *Amgad R Rezk* [£], *Joseph Halim* [§], *Per O. Å. Persson*[§], *Leslie Y Yeo* [£]*, *Johanna Rosen*[§]*

[§] Department of Physics, Chemistry, and Biology (IFM) Linköping University, SE-581 83 Linköping, Sweden.

[£] Micro/Nanophysics Research Laboratory, RMIT University, Melbourne, Victoria 3000, Australia.

[‡] These authors contributed equally to this work.

[*] Correspondence to: leslie.yeo@rmit.edu.au , johanna.rosen@liu.se.

Supplementary Text

Estimation of number of devices required for synthesis of 1 kg MXene per week.

Ti_3AlC_2 MAX Phase: 100 mg in 20 ml solution

MXene output: ~ 12 mg

Number of devices: 1

Flow rate: 0.2ml/min (*i.e.* 20 ml ~ 100 minutes)

The data above implies 12 mg MXene in 100 minutes for one device, which equals 0.173 g in 1 day.

For 830 devices, we will then produce 143.5 g /day. In a week that equals to 1 kg of MXene.

The level of safety of the SAW- and LiF-based method for producing $Ti_3C_2T_z$ from Ti_3AlC_2:

The input and the output of the technique are acid-free. The input comprises LiF (salt), MAX phase (Ti_3AlC_2) and water. The output comprises MXene ($Ti_3C_2T_z$), unetched MAX phase (Ti_3AlC_2), LiF residual particles, and water. During the process "*in situ* HF" is created with a maximum concentration of 0.1%, which is at the lower limit for the least hazardous HF concentration according to https://www.prevent.se/amnesomrade/kemiska-risker/. The hazards and safety precautions are found below. This amount of "*in situ* HF" is formed during the process in an enclosed container, and when the process is stopped, the F ions are depleted. This is because the amount of F^- ions produced from the dissolution of LiF in water is not sufficient to convert all Al in the MAX phase to AlF_3 hydrates. Therefore, the outcome of the process is acid free.

Fluoric acid (aqueous)

CLP-Classification

H300	Acute Tox. 2 - Acute toxicity
H310	Acute Tox. 1 - Acute toxicity
H314	Skin Corr. 1A - Skin corrosion/irritation
H318	Eye Dam. 1 - Serious eye damage/eye irritation
H318	Eye Dam. 1 - Serious eye damage/eye irritation
H330	Acute Tox. 2 - Acute toxicity

CLP-Marking

Concentration range

0.1% - 0.25%

Hazard pictograms

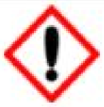

Signal word

VARNING

Hazard statements

H319	Causes serious eye irritation

Precautionary statement - Prevention

P264	Wash ... thoroughly after handling.
P280	Wear protective gloves/protective clothing/eye protection/face protection.

Precautionary statement - Response

P337+P313	If eye irritation persists: Get medical advice/attention.

Remarks

Harmonised classification according to (EG) nr 1272/2008.

Figure S1: Safety data sheet for 0.1-0.25 % conc. HF.

Electrochemical Characterization

Figure S4A presents the cyclic voltammogram profile of $Ti_3C_2T_z$ collected at 10 mV s^{-1} in 1 M H_2SO_4 electrolyte. Redox peaks can be identified in the CV profile, with both the oxidation peak and the reduction peak located at -0.55 V showing a highly reversible redox reaction. The open circuit voltage (OCV) was stable at 0.3 V. The electrodes used for current collection were glassy carbon, having the ability to suppress the hydrogen evolution reaction, with a wide voltage window of 0.9 V. The charge storage mechanisms were studied for the $Ti_3C_2T_z$ electrode through the CV currents collected at different scan rates, a method reported by Wang *et al.* ,[23]that relates the sweep rates and collected current through the $i= av^b$ formula, in which the b value is the slope of the log I *versus* log V curve. The storage mechanism is diffusion limited if the b value is ~0.5, while a capacitive storage mechanism is dominant for a b value of 1. As shown in Fig. S4A, the b value is close to1 at different scan rates. Figure S4B shows the Nyquist plot, and the observed straight vertical line in the low frequency region is an indication of pure capacitive behavior for the $Ti_3C_2T_z$ electrode. From the real axis, the equivalent series resistance (ESR) was 0.08 $\Omega.cm^2$. Both electrochemical stability and Coulombic efficiency are decisive factors in the evaluation of any energy storage system. By charging and discharging at a current density of 10 A g^{-1} for 10,000 cycles, the $Ti_3C_2T_z$ electrode (three-electrodes system) shows 92% stability and almost 100% Columbic efficiency (see Figure S4C), demonstrating outstanding long-life electrochemical stability. In the inset, the symmetric shape of the galvanostatic cycling profile is shown, being maintained after 10,000 cycles with only a very insignificant reduction in the capacitance. The CV profiles for a 2 μm film (see Fig. S4D) shows that the MXene charging mechanism is more or less maintained for different scan rates. Furthermore, a high-rate performance was observed. Figure S2E represents the method to calculate the b-value fitting parameter. The symmetrical behavior in the charge-discharge curves at various current densities, shown in Fig. S4F, confirms the high electrochemical reversibility.

For the sake of comparison between the produced MXene from the mild conventional etching (F_1) and the SAW technique (F_2), we prepare Ti_3C_2 MXene by mild conventional etching (12 M HCl and 5M LiF) and filter a film from an unwashed colloidal solution. This allows a more direct comparison to the results from the film originating from the SAW technique.

The XRD patterns in Fig S6a show that both the F_1 and F_2 films contain residual LiF particles. The electrochemical performance of a washed and unwashed film from conventional mild synthesis is 230 and 210 Fg^{-1}, respectively, *i.e.*, about 8 % reduction when washing is omitted. This is close to within the experimental error range.

A comparison between the F_1 and F_2 films is shown in Figure S6b indicating that the film originating from the SAW technique has about 14% lower capacitance at low scan rates compared to the conventionally derived material, though this difference decreases with increasing scan rate. It should be stressed that the initial work on SAW-facilitated MXene is produced for proof of concept, and the slight difference may at least in part be due to changes in the electrode materials (density, thickness, sheet size, *etc*.)

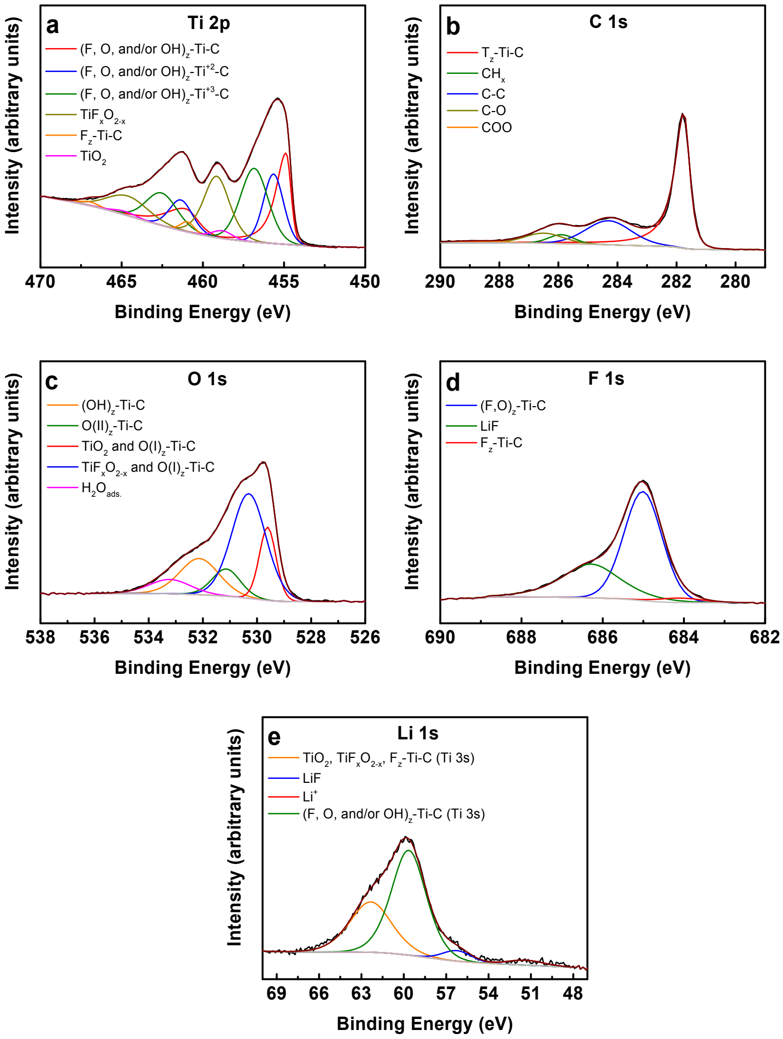

Figure S2. XPS spectra with curve-fitting for $Ti_3C_2T_z$ of the following regions: (A) Ti 2p, (B) C 1s, (C) O 1s, (D) F 1s and (E) Li 1s. Details for the labeled species are tabulated in Table S1.

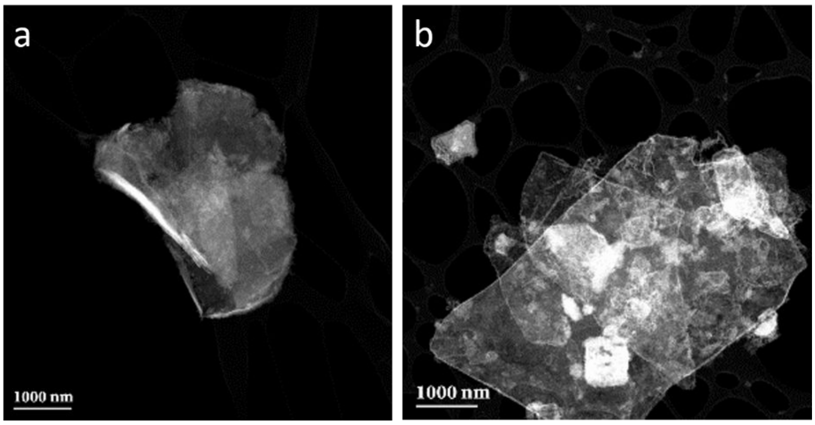

Figure S3. TEM analysis showing as prepared Ti_3C_2 single MXene sheets (> 1 μm).

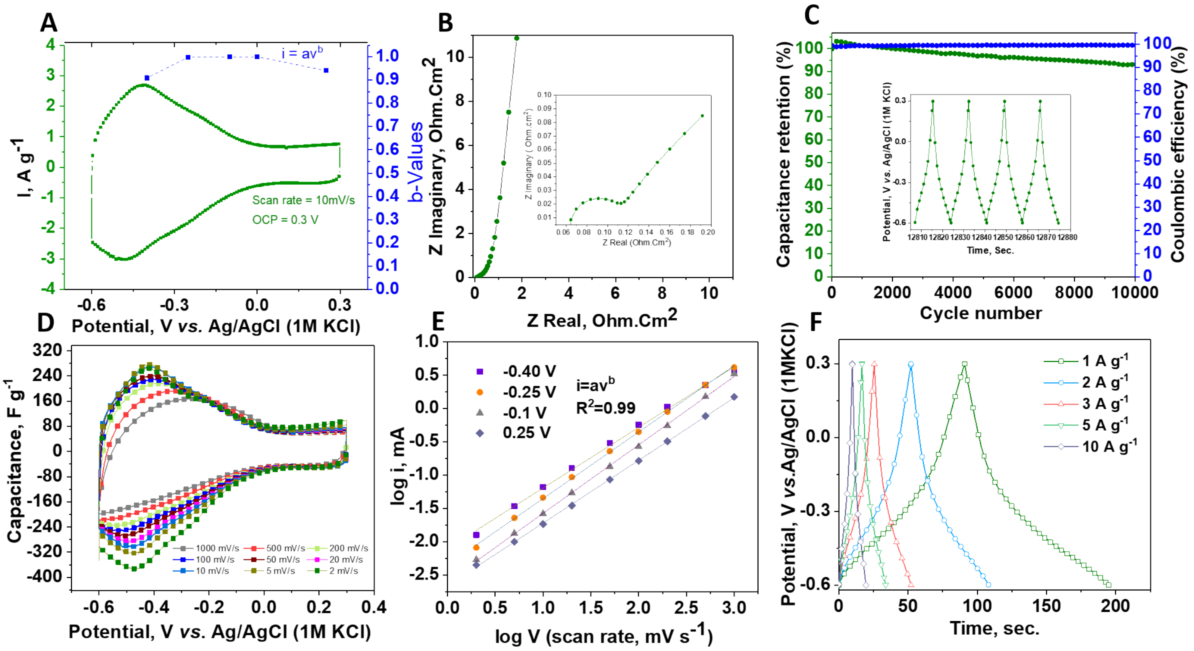

Figure S4. Electrochemical behaviour for a freestanding $Ti_3C_2T_z$ MXene film in 1 M H_2SO_4 *vs*. Ag/AgCl in 1M KCl. **(A)** Cyclic voltammetry profile measured at 10 mV S^{-1}. The b-values (blue symbols) are the slope of the logarithmic anodic current peaks against the logarithmic scan rates (2–1000 mV s^{-1}). **(B)** Nyquist plots of MXene collected *via* EIS; the inset shows the values in the low frequency region. **(C)** Capacitance retention and Columbic efficiency test for a 2 µm film electrode; the inset shows the galvanostatic charge/discharge at 10 A g $^{-1}$. **(D)** Normalized cyclic voltammetry profile measured at 2–1000 mV S^{-1} for the 2 µm film. **(E)** Logarithmic anodic current peaks *vs.* logarithmic scan rates (2–1000 mV S^{-1}) at different voltage windows for the 2 µm electrode. **(F)** Galvanostatic cycling at 1, 2, 3, 5 and 10 A g $^{-1}$.

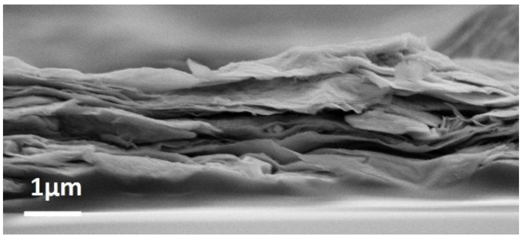

Figure S5. SEM image showing a cross-section of a $Ti_3C_2T_z$ MXene film of approximately 2 μm thickness used for electrochemical characterization.

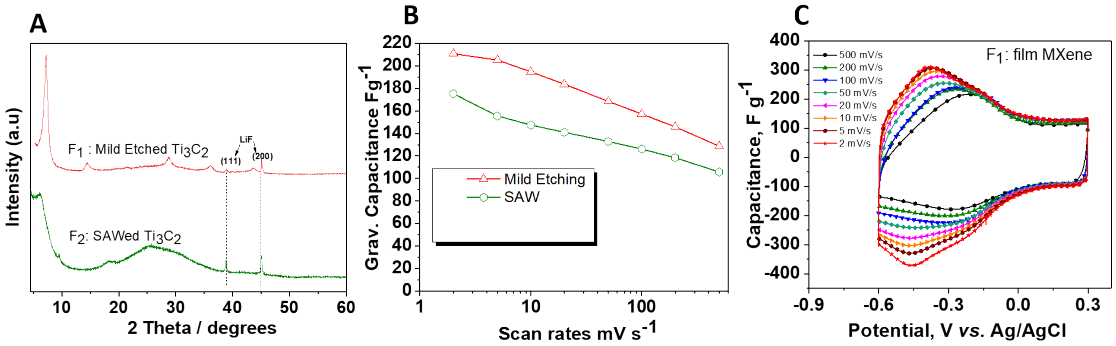

Figure S6. (A) XRD pattern for a free-standing film of $Ti_3C_2T_z$ MXene from Mild etching (F1) and from the SAW techniques (F2), without HCl and LiCl treatment to remove LiF residuals. **(B)** Gravimetric capacitance of $Ti_3C_2T_z$ MXene films in 1 M H_2SO_4 *vs*. Ag/AgCl in 1M KCl. **(C)** Cyclic voltammetry of the F_1 film at various scan rates.

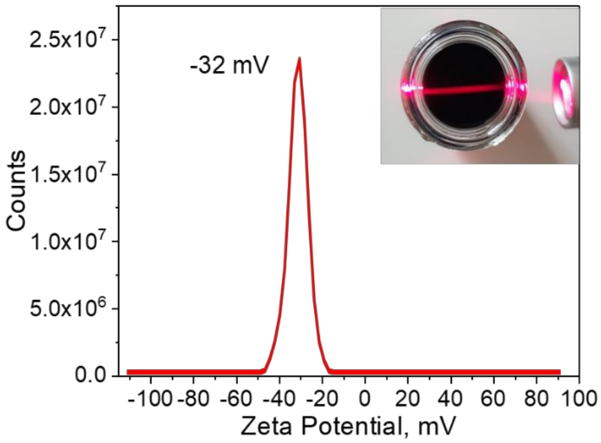

Figure S7. Zeta potential measurements of $Ti_3C_2T_z$ MXene sheets produced by the SAW technique, and a digital photo (figure inset) of the MXene colloids clearly showing a discernible Tyndall scattering effect.

Table S1. Summary of global atomic percentages obtained from the high resolution XPS spectra of the following regions: Ti 2p, C 1s, O 1s, F 1s, Al 2p and Cl 2p of a $Ti_3C_2T_z$ freestanding film.

Elements	Ti at. %	C at. %	O at.%	F at. %	Cl at. %	Li at. %	Al at. %
$Ti_3C_2T_z$	31.5±0.3	28.4±0.5	19.5±0.8	10.9±0.2	1.5±0.1	7.9±0.2	0.3±0.2

Table S2. XPS peak fitting results of $Ti_3C_2T_z$ for the following regions: Ti 2p, C 1s, O 1s, F 1s and Li 1s.

Region	BE [eV][a]	FWHM [eV][a]	Fraction	Assigned to	Reference
Ti 2p	454.9 (461.1)	0.7 (1.3)	0.22	(F, OH, and/or O)-Ti-C	(1,2,3)
	455.7 (461.3)	1.4 (2.1)	0.20	(F, OH, and/or O)-Ti^{2+} -C	
	456.8 (462.5)	2.0 (2.3)	0.29	(F, OH, and/or O)-Ti^{3+}-C	
	458.7 (464.7)	1.5 (3.7)	0.03	TiO_2	
	459.2 (464.7)	1.8 (2.6)	0.22	TiF_xO_{2-x}	
	461.0 (467.0)	1.7 (2.7)	0.04	F_z-Ti-C	
C 1s	281.8	0.5	0.61	T_z-Ti-C	(1,2,3)
	284.3	1.8	0.24	C-C	
	285.9	1.0	0.05	CH_x	
	286.5	1.5	0.08	C-O	
	288.8	2.0	0.02	COO	
O 1s	529.6	0.7	0.16	TiO_2 and $O(I)_z$-Ti-C	(1,2,3)
	530.3	1.5	0.47	TiF_xO_{2-x} and $O(I)_z$-Ti-C	
	531.1	1.2	0.10	$O(II)_z$-Ti-C/OR[b]	
	532.1	1.7	0.19	$(OH)_z$-Ti-C/OR[b]	
	533.2	1.8	0.08	H_2O_{ads}/OR[b].	
F 1s	684.1	1.3	0.03	$(F)_z$-Ti-C	(1,2,3)
	685.0	1.1	0.64	$(F, O)_z$-Ti-C	(1,2,3)
	686.3	1.7	0.33	LiF	(4,5)

Li 1s	51.4<br>56.2<br>59.6<br>62.3	3.0<br>2.4<br>2.9<br>3.5	0.37<br>0.63	$Li^{=}$ (Li 1s)<br>LiF (Li 1s)<br>(F, O, and/or OH)$_z$-Ti-C (Ti 3s)<br>TiO_2, TiF_xO_{2-x}, F_z-Ti-C (Ti 3s)	(4,5)

[a] Values in parentheses correspond to the $2p_{1/2}$ component.

[b] OR denotes the organic compounds that are present due to atmospheric surface contamination.

Note: the TiO_2 and TiF_xO_{2-x} species are formed during exposure of the sample to the ambient during transfer to the XPS (the formation of such oxides is common for MXene washing and/or exposure to the ambient).[5]

Table S3. List of various species assigned by XPS analysis.

Species Names	What does it belong to
(F, OH, and/or O)-Ti-C, (F, OH, and/or O)-Ti^{2+}-C and (F, OH, and/or O)-Ti^{3+}-C	Ti belonging to MXene compound with various oxidation states and terminated by F, O and/or OH surface groups
TiO_2, and TiF_xO_{2-x}	Surface oxides and oxyfluorides usually found on the surface of the sample due to its contact with oxygen from the air
F_x-Ti-C	MXene terminated by F surface group without the influence of other adsorbates or surface groups
T_z-Ti-C	C belonging to the MXene compound, where the MXene is terminated by F, O and/or OH surface groups
C-C, CH_x, C-O, and COO	Hydrocarbons not belonging to the MXene compound due to surface contamination and/or during washing
$O(I)_z$-Ti-C	Oxygen surface termination bridging to Ti atoms belongs to the MXene structure
$O(II)_z$-Ti-C	Oxygen surface termination occupying the FCC site belongs to the MXene structure
H_2O_{ads}	Adsorbed water on the MXene sheets

$(F, O)_z$-Ti-C	MXene terminated by F surface group with the influence of other adsorbates or surface groups
LiF	Unwashed LiF compound present from the etching solution
Li^+	Li ions intercalated between the MXene sheets

Table S4. Gravimetric and volumetric capacitance for a $Ti_3C_2T_z$ electrode.

Scan rate	Gravimetric capacitance, F g^{-1}	Volumetric capacitance, F cm^{-3}
2	174	470
5	156	420
10	147	398
20	141	381
50	133	359
100	126	340
200	118	320
500	106	286
1000	93	250

References:

1. Halim, J.; Cook, K. M.; Naguib, M.; Eklund, P.; Gogotsi, Y.; Rosen, J.; Barsoum, M. W., X-Ray Photoelectron Spectroscopy of Select Multi-Layered Transition Metal Carbides (MXenes). *Applied Surface Science* **2016,** *362*, 406-417.
2. Ren, C. E.; Zhao, M.-Q.; Makaryan, T.; Halim, J.; Boota, M.; Kota, S.; Anasori, B.; Barsoum, M. W.; Gogotsi, Y., Porous Two-Dimensional Transition Metal Carbide (MXene) Flakes for High-Performance Li-Ion Storage. *ChemElectroChem* **2016,** *3*, 689-693.
3. Dall'Agnese, Y.; Lukatskaya, M. R.; Cook, K. M.; Taberna, P.-L.; Gogotsi, Y.; Simon, P., High Capacitance of Surface-Modified 2D Titanium Carbide in Acidic Electrolyte. *Electrochemistry Communications* **2014,** *48*, 118-122.

4. Verger, L.; Natu, V.; Ghidiu, M.; Barsoum, M. W., Effect of Cationic Exchange on The Hydration and Swelling Behavior of $Ti_3C_2T_z$ Mxenes. *The Journal of Physical Chemistry C* **2019,** *123*, 20044-20050.
5. J. Halim, I. Persson, P. Eklund, P. O. Å. Persson, J. Rosen, Sodium Hydroxide and Vacuum Annealing Modifications of The Surface Terminations of a Ti_3C_2 (Mxene) Epitaxial Thin Film. *RSC Adv.* **2018,** 8, 36785-36790.
